# Cardiovascular magnetic resonance phase contrast imaging

**DOI:** 10.1186/s12968-015-0172-7

**Published:** 2015-08-09

**Authors:** Krishna S. Nayak, Jon-Fredrik Nielsen, Matt A. Bernstein, Michael Markl, Peter D. Gatehouse, Rene M. Botnar, David Saloner, Christine Lorenz, Han Wen, Bob S. Hu, Frederick H. Epstein, John N. Oshinski, Subha V. Raman

**Affiliations:** Ming Hsieh Department of Electrical Engineering, University of Southern California, 3740 McClintock Ave, EEB 406, Los Angeles, California 90089-2564 USA; Department of Biomedical Engineering, University of Michigan, Ann Arbor, MI USA; Mayo Clinic, Rochester, MN USA; Department of Radiology, Northwestern University, Chicago, IL USA; Cardiovascular Biomedical Research Unit, Royal Brompton Hospital, London, UK; Cardiovascular Imaging, Imaging Sciences Division, Kings’s College London, London, UK; Department of Radiology and Biomedical Imaging, University of California San Francisco, San Francisco, CA USA; Center for Applied Medical Imaging, Siemens Corporation, Baltimore, MD USA; Imaging Physics Laboratory, National Heart Lung and Blood Institute, Bethesda, MD USA; Palo Alto Medical Foundation, Palo Alto, CA USA; Departments of Radiology and Biomedical Engineering, University of Virginia, Charlottesville, VA USA; Departments of Radiology and Biomedical Engineering, Emory University School of Medicine, Atlanta, GA USA; Division of Cardiovascular Medicine, The Ohio State University, Columbus, OH USA

**Keywords:** CMR flow imaging, Phase contrast, Valvular disease, Congenital defects

## Abstract

Cardiovascular magnetic resonance (CMR) phase contrast imaging has undergone a wide range of changes with the development and availability of improved calibration procedures, visualization tools, and analysis methods. This article provides a comprehensive review of the current state-of-the-art in CMR phase contrast imaging methodology, clinical applications including summaries of past clinical performance, and emerging research and clinical applications that utilize today’s latest technology.

**Electronic supplementary material:**

The online version of this article (doi:10.1186/s12968-015-0172-7) contains supplementary material, which is available to authorized users.

## Introduction

The assessment of blood flow parameters is important to the study of cardiovascular function and to the clinical evaluation of cardiovascular disease. Evaluation of the heart valves requires identification and quantification of stenoses and regurgitation, and congenital cardiac abnormalities require identification and quantification of shunt flow. Building on early developments in NMR and MRI that enabled the measurement of flow velocity and velocity distributions [1–3], applications of MRI to flow assessment in cardiovascular disease began in the mid 1980s [4–9], first to the assessment of flow in the heart, and later to the large vessels (e.g., aorta, carotids). Many technological innovations have led to the ability to reliably quantify regurgitant and shunt flow volumes, visualize 3D time-resolved flow patterns, and assess coronary flow reserve, wall shear stress, and turbulence. Cardiovascular MR (CMR) has become an important complement to echocardiography in the clinic, and in the evaluation of congenital heart disease (CHD) in centers with the required expertise. In cardiovascular research, CMR flow imaging is enabling a range of emerging applications such as assessment of vessel compliance and myocardial motion.

This article describes the current state-of-the-art in CMR flow imaging methods and applications. The first section covers methodology and technical issues including pulse sequences, calibration, visualization, and analysis. This section includes *practical* advice appropriate for both research and clinical users. The second section covers two major clinical applications, valvular disease and congenital disease. These applications rely on imaging methods that have been thoroughly validated, and are feasible with commercially available pulse sequences and analysis software. The third section covers emerging applications and technologies that have the potential to impact clinical medicine and/or basic cardiovascular research.

This article will also address several common questions: What is the best way to avoid or correct velocity offsets and other artifacts? What is the impact of high-field systems and parallel imaging? What visualization tools have been found to be the most informative?

## I. Methodology

### Physical principles and imaging methods

MRI is a phase-sensitive modality that can encode information about velocity into the phase of the detected signal. Flow volume is typically measured by obtaining a thin, cross-sectional image of the vessel of interest using phase contrast methods that are sensitized to through-plane velocity [6, 10–12]. The vessel lumen is covered by a set of pixels. We calculate the flow *Q*_*i*_ through pixel *i* by forming the product

1 $${Q}_i={a}_i\times {v}_{\perp i}$$

where *v*_⊥*i*_ is the measured, perpendicular component of the fluid velocity through the *i*th pixel, which has area *a*_*i*_. The measured velocity *v*_⊥*i*_ is in practice a weighted average of velocities within the pixel. The total flow *Q* through the vessel is then calculated by summing over the *N* pixels that cover the vessel lumen in the image. The pixel areas are typically identical (i.e., *a*_*i*_ = *a*), and can be removed from the summation:

2 $${Q}_{tot}={\displaystyle \sum_{i=1}^N{Q}_i={\displaystyle \sum_{i=1}^N{a}_i{v}_{\perp i}}=a{\displaystyle \sum_{i={1}_i}^N{v}_{\perp i}}=(Na)}\left[\frac{1}{N}{\displaystyle \sum_{i=1}^{N}{v}_{\perp i}}\right]=A\left\langle {v}_{\perp}\right\rangle$$

Equation 2 shows that the net flow is given by the product of the area of the vessel lumen *A* = *Na* and the average perpendicular component of velocity 〈*v*_⊥_〉 over the vessel lumen. If 〈*v*_⊥_〉 is measured in units of cm/s and *A* is measured in units of cm^2^, then to express *Q* in units of mL/min requires a multiplicative conversion factor of 60 s/min.

#### Phase-contrast velocity mapping

**Fig. 1:**
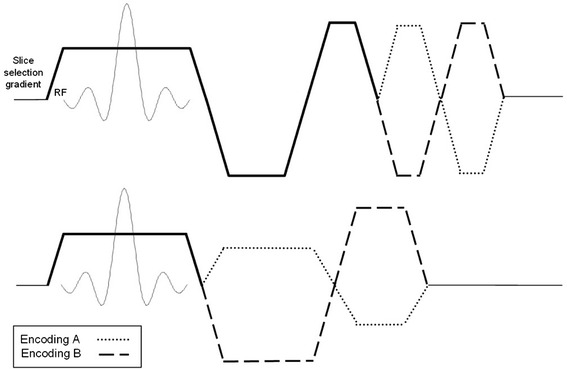
MR images are sensitized to measure the through-plane component of velocity by applying a flow-encoding gradient to the slice-selection axis of the pulse sequence. The flow encoding gradient can be added as *(top)* a bipolar pair to a flow-compensated slice selection waveform, or *(bottom)* to reduce minimum TE, combined with other gradient lobes

Consider a single slice acquired at a single cardiac phase. To obtain the velocity map (i.e., an image where pixel intensity is proportional to *v*_⊥*i*_), a flow-encoding gradient is applied along the slice-selection direction of the imaging pulse sequence, after the excitation but before the readout. Figure 1 shows representative pulse sequence diagrams for the slice-selection gradient axis. The flow-encoding gradient can be applied as an additional pair of toggled, bipolar gradient lobes. Alternatively, those lobes can be combined with slice selection gradient waveforms, in order to reduce the minimum echo time. The typical acquisition is 2DFT gradient recalled echo (GRE) with either a full echo or a partial echo in the readout direction. Other k-space acquisition trajectories, including echo planar [13, 14], spiral [15, 16] and radial [17], have been employed to improve acquisition speed or to reduce flow artifacts.

Spins that flow along the direction of the flow-encoding gradient accumulate phase *ϕ*_*i*_ = *γm*_1_*v*_⊥*i*_ + *ϕ*_0i_, where *γ* is the gyromagnetic ratio in units of rad/s/T, and *m*_1_ is the first moment of the flow-encoding gradient (i.e. the time-weighted sum of the gradient area, *m*_1_ = ∫ *G*(*t*)*tdt*). The term *ϕ*_0i_ represents all contributions to the MR phase that are not related to flow. This includes phase due to off-resonance, complex RF coil receive sensitivities, and the effect of imperfect echo centering in the readout window. When the gradient waveform is toggled between two shapes *A* and *B*, as indicated in Fig. 1, the change in first moment is given by *m*_1*A*_ and *m*_1*B*_ so that for each pixel:

3 $$\Delta {\phi}_i={\phi}_{iA}-{\phi}_{iB}=\gamma \Delta {m}_{\perp }{v}_{\perp i}$$

To a good approximation, the confounding contribution to the phase *ϕ*_0*i*_ drops out of Eqn. 3, while the resulting expression for Δ*ϕ* remains proportional to *v*_⊥*i*_. As discussed in more detail later, some residual contributions to *ϕ*_0*i*_, notably those from gradient eddy currents and from the concomitant field, are not completely canceled by the subtraction operation in Eqn. 3 and usually require further correction with post-processing.

Two complete sets of raw image data are acquired, *A* and *B*, with a difference in gradient first moment Δ*m*_1_. A phase difference reconstruction [18, 19] is then applied to the two raw data sets to obtain an image where the intensity of the *i*th pixel is proportional to the velocity *v*_⊥*i*_. The sign of Δ*ϕ*_*i*_ represents the flow direction. A magnitude image *M* is reconstructed from the same raw data, either by averaging the reconstructed magnitude images *M*_*A*_ and *M*_*B*_, or by using *M*_*A*_ exclusively, provided acquisition *A* is flow-compensated (i.e., *m*_1*A*_ = 0). Because the gyromagnetic ratio constant *γ* and the factor Δ*m*_1_ are known, we can quantitatively extract the value of *v*_⊥*i*_ using Eqn. 3.

#### Acquisition considerations

A single slice location is sufficient to quantify flow through a vessel using the method suggested by Eqn. 2. The slice is prescribed in an oblique plane so that it perpendicularly intersects the vessel of interest. Although we should always strive for perfect slice orientation, for sufficiently thin slices (e.g., 3 mm-thick or less), the measured flow rate is relatively insensitive to small angular deviations from true perpendicular (e.g., *β* < 20^o^). This insensitivity arises because, to first order, errors in two multiplicative factors in Eqn. 2 cancel: 〈*v*_⊥_〉 is reduced by a factor of *cosβ*, while the area *A* is increased by a factor of (*cosβ*)^−1^ [20, 21].

A time-resolved or multiphase acquisition is required to accurately quantify pulsatile, arterial flow. To enable the acquisition of a slice location within a breath hold (e.g., approximately 18 heartbeats), a segmented [22] acquisition is often used. For example, if we acquire 6 phase encoding lines (views) per segment, then during the first R-R interval, flow encodings *A* and *B* for views 1-6 are repeatedly acquired to yield approximately 10-20 cardiac phases, depending on the patient’s heart rate and details of the pulse sequence and the performance specifications of the scanner hardware. In the second R-R interval, data for views 7-12 are acquired, and so on, until the entire k-space is filled for both flow encodings *A* and *B*. The resulting multiphase phase contrast data are often reconstructed using view sharing to increase the apparent temporal resolution [23–25].

#### Flow-related aliasing

The measured velocity component *v*_⊥*i*_ provides an excellent approximation to the true, average velocity component within the voxel [20, 26] unless flow-related aliasing occurs. The *aliasing velocity* is an operator-selected parameter of the phase contrast pulse sequence. It is often denoted by *v*_*enc*_ or VENC, which is the maximum encoded velocity and is measured in units of cm/s. Provided the average perpendicular component of velocity within a voxel lies within the range, − *VENC* < |*v*_⊥_| < *VENC* the pixel intensity in the phase difference image remains linearly proportional to *v*_⊥*i*_, as indicated by Eqn. 3. Because the phase difference reconstruction returns a value in the range −*π* ≤Δ*ϕ*_*i*_ ≤ +*π*, VENC is related to the change in first moment by:

4 $$VENC=\frac{\pi }{\upgamma \Delta {m}_{\perp }}$$

5 $${v}_{\perp i}= VENC\frac{\Delta {\phi}_i}{\pi }$$

**Fig. 2:**
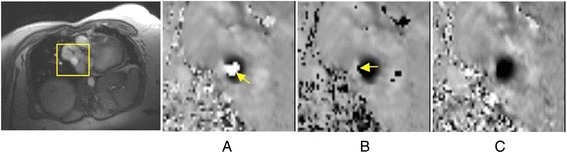
Difficulty where wraparound is not an “island” in the flow: **a** Peak flow through a mildly narrowed pulmonary vein showing velocity aliasing of black into white (arrow) at VENC 80 cm/s. **b** Unwrapping this aliasing was uncertain in partial-volume pixels neighboring the reverse flow channel (arrow) offset 50 cm/s. **c** The same plane acquired at VENC 130 cm/s showed true forward flow in black and the reverse flow channel at its left on the image. (provided by Dr. Sylvia Chen, Royal Brompton Hospital, London, UK)

When |*v*_⊥*i*_| exceeds VENC, velocity aliasing will occur. That means that velocities in excess of ± VENC will be mapped (i.e., aliased) erroneously to velocities within the range of ± VENC. Besides misrepresenting speed, flow-related aliasing can also result in an artifactual reversal of the displayed flow direction. Unfortunately, the aliasing velocity cannot be set arbitrarily high because that incurs a signal-to-noise ratio (SNR) penalty. Provided that |*v*_⊥_| < *VENC*, the SNR of the phase difference image is given by

6 $$SN{R}_{\Delta \phi}\propto SN{R}_M\times \frac{v_{\perp }}{VENC}$$

where SNR_M_ is the signal-to-noise ratio of the reconstructed magnitude image. Notice that the phase SNR is proportional to signal magnitude and velocity, but inversely proportional to VENC. A low VENC results in higher phase SNR, however, if too low a value of VENC is selected, unwanted flow-related aliasing occurs. Optimising an acquisition with low VENC setting may require several acquisitions until the peak velocity is free of aliasing. It is possible to acquire images at multiple VENCs within one breath-hold to assist this iterative optimization (i.e., VENC Scout) but an acquisition at reduced spatial and temporal resolution may miss some aliasing, though aggressive scan-time acceleration may be used to alleviate this [27]. In some cases, flow-related aliased images can be salvaged with a post-processing technique called phase-unwrapping [28–30]. Typically, however, some SNR is intentionally sacrificed by selecting the value of VENC to be sufficiently high to avoid aliasing in most patients, e.g., 200 cm/s to measure healthy aortic flow. If resulting images still have unacceptable flow-related aliasing and phase unwrapping methods are not available, the acquisition can be repeated with a higher value of VENC, as illustrated in Fig. 2.

#### Fourier velocity encoding

In some cases, it is desirable to measure the distribution of velocities within a pixel, rather than only the weighted average. This can be accomplished with Fourier velocity encoding [3], where the two flow encoding values mentioned earlier, *A* and *B*, are replaced by a longer series (e.g., 8 or 16 values) of flow encoding steps separated by constant increment of the first moment Δ*m*_1_. The resulting data are then reconstructed with a discrete Fourier transform instead of a phase difference method. At each pixel, this procedure yields a set of images, each sensitive to velocity within a specific range, or bin.

One advantage of Fourier velocity encoding is that by examining the zero velocity image, we can measure the stationary tissue contribution to the signal for a particular pixel. This is particularly useful at the boundary of the vessel lumen, where pixels cover both flowing blood and stationary tissue. As discussed in more detail later, such partial volume effects are a common source of systematic error when using standard MR methods to quantify flow.

The main disadvantage of Fourier velocity encoding, however, is that it increases the acquisition time by a factor of *N*/2, where *N* is the number of velocity encoding steps, compared to a standard phase contrast measurement with 2 flow encoding steps. There have been attempts at compensating by using faster acquisition strategies [31, 32]. However, because of this acquisition time penalty, Fourier velocity encoding has not been widely used. Instead, as discussed later, the most popular countermeasure against partial volume errors is simply to increase the spatial resolution using standard velocity mapping methods. Fourier velocity encoding, however, has found application with 1D pencil beam excitation imaging [33] (discussed further below), which is intrinsically fast.

#### Measuring the velocity vector

In some cases, it is desirable to measure the complete velocity vector ${\overline{v}}_i$ for each pixel *i*, rather than only its perpendicular component *v*_⊥*i*_. Although this complete vector measurement is not necessary to measure flow through a vessel, it is required for other applications such as tissue velocity mapping, as discussed later. Measurement of the velocity vector requires a minimum of four flow-encoded measurements [34], which we will label *A*, *B*, *C*, *D*. Often *A* is acquired with flow compensation on the slice selection and frequency encoding axes. This involves adding gradient pulses that null the 0^th^ and 1^st^ moment of the gradient waveforms between excitation and data collection. Then the first moment is changed by Δ*m*_1_ on one axis at time for the *B*, *C*, and *D* acquisitions. For example, we could set the first moment on the frequency encoding axis to be Δ*m*_1_ for encoding *B;* the first moment on the phase encoding axis to be Δ*m*_1_ for encoding *C,* and similarly for the slice selection axis for encoding *D*. Using acquisition *A* as a common phase reference, three independent phase difference reconstructions and Eqn. 3 yields three separate velocity maps, each of which is sensitized to flow in one orthogonal direction. Calling the three velocity maps *v*_*xi*_, *v*_*yi*_ and *v*_*zi*_, the vector velocity for pixel *i* is given by $\overline{v_i}=\left({v}_{xi},{v}_{yi},{v}_{zi}\right)$. If desired, a map of the flow speed can be calculated from $\left|\overline{v_i}\right|=\sqrt{{v}_{xi}^2+{v}_{yi}^2+{v}_{zi}^2}$.

#### 4D flow CMR

3D spatial encoding offers the possibility of isotropic high spatial resolution and thus the ability to measure and visualize the temporal evolution of complex flow and motion patterns in a 3D volume, without any restrictions to predefined imaging planes. In this context, ECG synchronized 3D phase contrast CMR (PC-CMR) using 3-directional velocity encoding can be employed to detect and visualize global and local blood flow characteristics in targeted vascular regions [35, 36]. A number of recent methodological improvements permit the accelerated acquisition of such data within scan times of the order of 5-10 minutes. The number of potential applications of 4D flow CMR is growing, as covered by a number of recent review articles [37–43].

#### Pulse sequence details and vendor acronyms

While often not stated explicitly, PC-CMR acquisitions are generally RF-spoiled [44] to suppress multiple-TR signal pathways. In the absence of flow-induced intra-voxel dephasing (discussed below), RF-spoiled magnitude images have mixed T1 and spin-density weighting, and some T2* weighting depending on the TE. Several cycles of gradient dephasing across the voxel is needed to suppress ghost artifacts [45]. RF-spoiled 2DFT sequences have vendor names such as spoiled gradient echo (SPGR), fast low angle shot (FLASH), and T1-weighted fast field echo (T1-FFE). Since the PC-CMR sequence is typically interrupted at the end of each R-R interval while “waiting” for the next ECG trigger, the corresponding sequence names Fast SPGR, TurboFLASH, and TFE are sometimes used instead. Spiral phase-contrast sequences are not widely available, and in research papers are referred to as “spiral SPGR” or simply “spiral”.

### Artifacts and calibration procedures

PC-CMR is susceptible to unique artifacts that may alter the qualitative visualization of flow, and introduce errors in the quantification of flow volume or other hemodynamic parameters. There is no single dominant source of error, however several smaller errors may combine to result in velocity or flow measurement errors on the order of 5 % to 10 % or more. The earliest work that validated PC-CMR methods [46–48] included descriptions of these artifacts, and more recent reviews can be found in [26, 49]. It is important to note that the relative importance of different sources of artifact depends on the type of measurement being made, e.g., peak velocity or volume flow, and the type of pulse sequence being used. This section specifically discusses artifacts in PC-CMR acquired with 2DFT gradient echo pulse sequences.

#### Velocity signal and velocity noise

As suggested by Eqn. 6, reliably strong blood signal is fundamental to PC-CMR. Exactly as in gradient-echo cine imaging, in PC-CMR cine imaging, the longitudinal magnetization of material in the slice is partially saturated by the repeated RF pulses, more so for long T1s such as blood. Inflow of unsaturated material into the image plane or volume produces bright signal [50]. For large volume imaging, there is little inflow often resulting in low steady-state signal, thus requiring the use of low flip angles or T1-shortening contrast agents [51].

The total signal within a voxel may also be reduced or entirely lost by dispersion caused by spatially non-uniform, motion-induced phase shifts within the voxel, also known as intra-voxel dephasing or intra-voxel phase dispersion. This can be reduced by using flow compensation, however, PC-CMR requires that at least one flow-encoded image be obtained and by definition this image cannot be flow-compensated. There is the potential for regions of weak blood signal in that image, whereas the “reference” image can be fully velocity-compensated, producing strong blood signal. The subtraction of the two phase images described by Eqn. 3 will be unreliable if either image has low magnitude. The noisy phase of a weak magnitude signal leads to erroneous pixels around jet flows, e.g., “edge spike” artifacts [52] or “salt and pepper noise” [53]. Most systems offer “balanced” or “symmetric” velocity encoding shared between the two images [54], which can reduce signal loss due to intravoxel phase dispersion.

Signal can also be lost when there is turbulence or unsteady motion on the timescale between excitation and echo (a few milliseconds). In such cases, much of the signal can be recovered by shorter duration of the gradient waveforms so that there is less time for incoherent flow-related phase dispersion to occur [52, 53, 55], perhaps ultimately using TE times on the order of 1 ms [56]. The shorter gradient pulses do imply that stronger gradient amplitudes are required, which partially counteract the strong benefit of shorter TE [48]. Image post-processing techniques can remove unreliable pixels [57], but such methods require experienced supervision to avoid unnecessary suppression of true velocity data. The potential for high noise in individual pixel velocity measurements is one of the reasons that peak velocity determination in stenoses should be made using several pixels [53].

The problem of low velocity SNR can also be addressed in post-processing using advanced denoising techniques. Several such methods are based on the assumption of incompressible flow, which in turn dictates that flow fields must be divergence-free. By fitting the measured velocity field to a set of divergence-free “basis” functions [58] or vector fields [59], a strictly divergence-free flow field with reduced noise is obtained. Ong et al. [60] recently proposed a related denoising technique that retains a non-divergence-free flow component, which may arise due to, e.g., partial volume and flow discretization. The problem of suppressing noise while retaining physically meaningful flow information is an active area of research.

Flow measurement will be impacted if the vessel boundary region-of-interest (ROI) includes pixels whose signals are only partially from flowing magnetization (“partial volume” effect). The consequence depends on the surrounding tissue signal strength compared to that of blood, which is increased by fresh inflow enhancement. If the blood *magnitude* is far brighter than the surrounding tissue, and if the ROI includes the *entire* border pixel, then there will be an over-estimation of the total flow. If the two magnitudes are similar and the ROI again includes the entire partial-volumed pixel, the total flow will be correct. However, given the image interpolation often applied, ROI software might not include the entire partial-volumed raw pixel. In other words, the error due to “partial volume” effects can depend on the implementation details of the ROI software as much as on the relative signal strength of blood and surrounding tissue. The dependence of blood signal strength on fresh inflow confounds any general rule about setting ROI drawing thresholds from magnitude images. Nevertheless, drawing ROIs on magnitude images is generally found to be easier than drawing on velocity images. Perhaps the most detailed study [61] concluded this difference was “almost negligible for clinical purposes” in a great vessel model even with plug or skewed velocity profiles causing high velocities and bright blood magnitude near the wall. In smaller vessels, to keep partial-volume error below 10 %, at least 16 pixels should cover the vessel [20, 21, 61]. Complex-difference processing [62] and paraboloid fitting [63] reduce flow measurement errors, but are subject to limitations such as the assumption of laminar flow. In addition to these considerations, it has recently been recognized that partial volume from perivascular fat can cause chemical shift-induced velocity measurement errors, which can be minimized by imaging with high receiver bandwidth and choosing the TE such that fat and (stationary) blood signal are in-phase [64].

#### Image artifacts

Fluctuations in flow velocity during a 2DFT acquisition can create ghosting artifacts along the phase-encoded direction [65, 66]. This could be due to cardiac variability of flow-related phase shifts in the raw data, which the image reconstruction then assumes were made by the spatial phase-encoding gradient pulses. Flow changes have been documented during large inspiratory breath-holds [67], end-expiratory breath holds [68, 69], and during free breathing [70, 71]. One common appearance is that of replication of blood vessels along the phase-encoded direction. The symmetric velocity encoding approach can also weaken these artifacts. Mis-triggering or variable delay in ECG-triggering exacerbates such artifacts because it increases cardiac phase variability between sections of raw data. Signal averaging in non-breath-hold PC-CMR can reduce ghosting arising from random and pseudo-random flow variability [72, 73].

The flow itself can also appear displaced, due to motion during the time differences between slice-excitation, velocity-encoding, phase-encoding, and frequency-encoding. These effects reduce with short TE [48, 74, 75], but can still be significant in the case of flow jets. For example, a 4 m/s post-stenotic jet moves 12 mm in 3 ms, which by current standards would be a short time between excitation and frequency encoding. This would result in a displacement of the visualized flow by up to 12 mm within the reconstructed image. The exact location of the displaced signal depends on the orientation of flow with respect to the phase and frequency encoding gradients [74], and may even end up outside of the vessel. Phase-encode velocity compensation, if correctly implemented, also reduces distortion of oblique flow [8, 76, 77], except for that due to acceleration [78].

Slice positioning near mobile cardiac valves especially for regurgitation measurement has to balance multiple inaccuracies including distal Windkessel, coronary flow, proximal valve motion and signal loss [79, 80], even if repeatability is precise [81]. Prospective [82] and retrospective [83] valve-plane tracking may offer improvements beyond what is currently available on commercial scanners.

Misalignment of the velocity-encoding gradient with respect to the flow of interest results in an underestimation of velocity by the cosine of the misalignment angle, as discussed previously. For peak velocity assessment, a “splayed” jet emerging from a narrow or irregularly shaped orifice may defy accurate velocity measurement [84]. Finally, truncation or Gibbs artifacts can affect flow measurements, but are most problematic only when there is another nearby vessel [85].

#### Velocity errors

After the subtraction operation to form the phase difference image (Eqn. 3) there are residual contributions to Δφ_i_, notably those from gradient eddy current effects [57], concomitant field (i.e., Maxwell) terms [86], and gradient field distortions [87]. The residual phase errors appear as a non-zero velocity in stationary tissue but they affect the entire image often with gradual spatial variation. If uncorrected, all three effects can significantly distort the measured velocities and flow volumes and can also result in distortion of 3D streamlines and 3D particle traces, described in the Flow Visualization section. Such phase offset errors exhibit a substantial increase with increasing distance from the isocenter of the MR system, with the concomitant field and gradient field distortion in particular varying super-linearly with distance. Even small systematic inaccuracies in measured velocity can propagate into larger errors when computing volume flow. Its consequences and corrections are reported in [88–90]. 2D PC-CMR measurements performed in single vessel segments at or near the isocenter of the magnet are relatively insensitive to these errors. For 2D or 3D PC-CMR with large anatomic coverage, however, correction is required. Increasing interest in CMR phase contrast imaging to quantify valvular heart lesions such as mitral regurgitation [91] warrant re-examination of elements in clinical scan protocols such as phantom scan or other approaches to correct for phase offsets that remain problematic on current-generation scanners.

Corrections may be derived from stationary tissue [57, 92], or derived from a stationary phantom acquired with identical slice and pulse sequence parameters. Alternatively, Giese et al. [93] recently demonstrated that baseline PC offsets can be measured directly using magnetic field probes, which obviates the need for potentially error-prone post-processing but requires specialized hardware. Similar measurements by Busch et al. [94] showed that PC offsets can vary significantly with the temperature of the gradient coil mount, indicating the need for thermal stabilization or dynamic offset correction. While background velocity offsets may be insignificant for peak velocity assessment, on some machines and protocols the baseline offset becomes significant due to the area and temporal summations used to calculate volume flows such as cardiac output. Even small parameter changes such as slice thickness or in-plane orientation may alter the offset, which can arise from small errors in the eddy current correction or the essential concomitant gradient correction [86]. Using the smallest possible value of VENC or varying VENC through the cardiac cycle [95] may reduce the offset error in comparison to the velocity phase shift, but this is not always the case; for example, a smaller value VENC can provoke a larger offset. Positioning the vessel of interest near isocenter, or more realistically in the z = 0 plane, minimizes the phase error from the concomitant field and generally assists with many other aspects of scanner performance. Furthermore, these background velocity offsets can be temporally stable between cine frames conferred by cine imaging in breath-hold or free-breathing sequences [96], which facilitates their identification and removal during post-processing when the flows of interest are pulsatile. However, large temporal variations can occur due to, e.g., respiratory navigators in prospective cardiac gating sequences.

### Impact of parallel imaging, high field systems, and constrained reconstruction

PC-CMR requires multiple image acquisitions with differing first gradient moments, which can lead to long acquisition times. PC-CMR therefore particularly benefits from acceleration via parallel imaging techniques, which rely on receive coil arrays to reconstruct images from only a subset of the complete k-space data. There are two “classic” parallel imaging techniques that are supported by vendors: sensitivity-encoded MRI (SENSE) and generalized autocalibrating partially parallel acquisitions (GRAPPA). SENSE [97] operates in image space, and is able to unwrap field-of-view aliasing by combining images from a number of receiver coils once the sensitivity profiles of the coils have been obtained. Therefore, SENSE reduces scan time by reducing the acquired field-of-view. GRAPPA [98], on the other hand, operates in k-space, and relies on the k-space profiles of multiple receiver coils to fill the gaps between sampled k-space lines. Both SENSE and GRAPPA are phase-sensitive and can be used to accelerate PC-CMR, and are supported by vendors. Note that the use of these techniques in PC-CMR does not differ fundamentally from their use in other applications.

The cost of speeding up image acquisition with SENSE or GRAPPA is local image noise enhancement, which generally worsens with increasing acceleration (or “reduction”) factor R, and improves with increasing number of receive coils. The speed-up factor achievable with SENSE and GRAPPA are comparable, and values of R = 2 to 3 have been reported in 2D and 4D PC-CMR imaging at 1.5 T [99–106]. Parallel imaging can also be combined with Cartesian or spiral EPI for real-time imaging [100, 107], for which similar acceleration factors have been reported. There are no strict criteria for setting the speed-up factor, and it is ultimately up to the user to select an R that results in acceptable noise and artifact levels for a given application.

The trade-off between R and noise/artifact levels becomes more favorable at higher fields, since the image SNR improves with increasing field strength, and because spatial coil sensitivity profiles become more independent (localized). In addition, 3D spatial encoding is particularly well-suited for parallel imaging since, for a given net acceleration factor, “sharing” the undersampling between the phase- and partition-encoding dimensions can lead to improved image quality compared to a 2D acquisition. For these reasons one can generally expect 4D flow imaging at high field to enable the highest speed-up factors [105, 106] [108–122]. Alternatively, for a given reduction factor R, moving to higher fields can produce smoother 3D flow streamlines, reduced noise-induced measurement bias, and improved image SNR which aids vessel segmentation [121].

Several groups have explored the possibility of speeding up image acquisition further by combining parallel imaging with advanced, often iterative and non-linear, constrained reconstructions that exploit spatio-temporal correlations or sparsity [123–134]. These research methods go under a variety of names, typically including keywords such as “k-t”, compressed sensing, and spatio-temporal sparsity/constraints/correlations. Spatio-temporal constraints can also be combined with non-Cartesian undersampled PC-CMR acquisitions such as 2D radial [135, 136], 3D radial (“PC-VIPR”) [137], and 3D stack-of-stars [138]. The role that these advanced acquisition and reconstruction schemes may be able to play in routine clinical practice is the subject of intense ongoing research.

### Image analysis and flow visualization

**Fig. 3:**
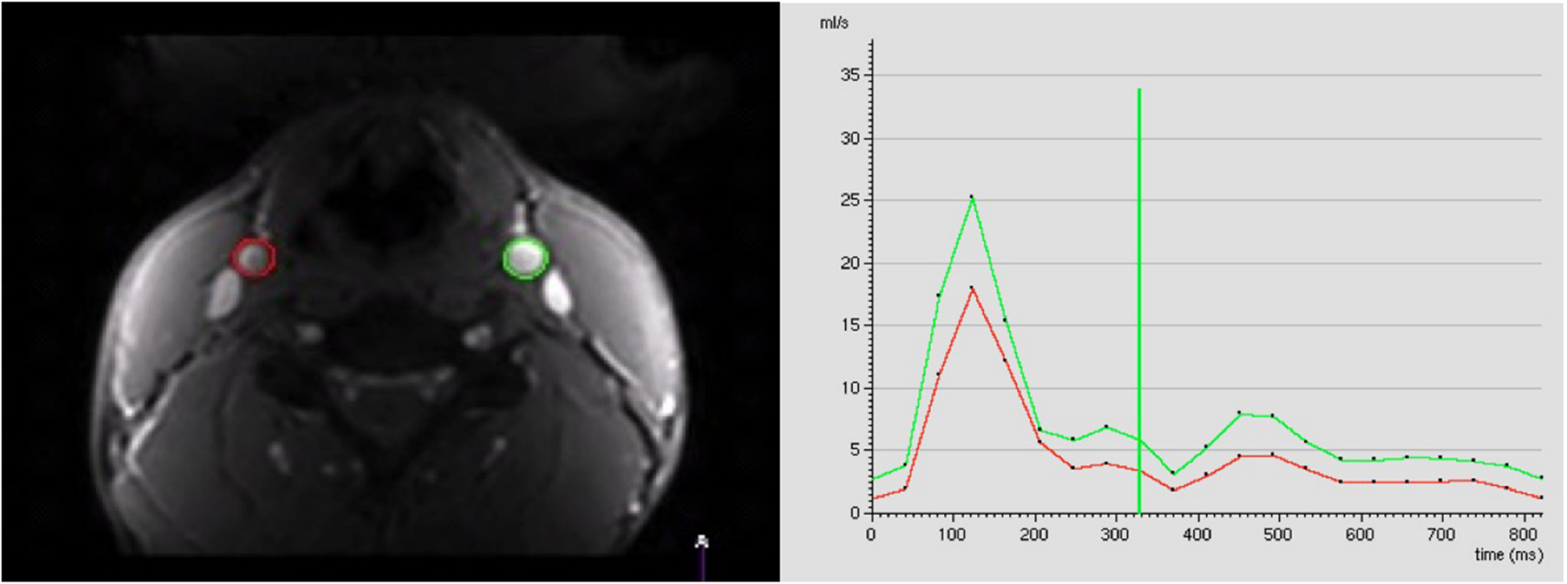
The left and right carotid arteries are outlined at each time frame on the phase or magnitude images using ROI’s (*left*). The instantaneous flow rate values, Q(t) are determined at each time frame. The flow rate from each time frame is plotted versus time in the cardiac cycle to yield flow curves (*right*)

**Fig. 4:**
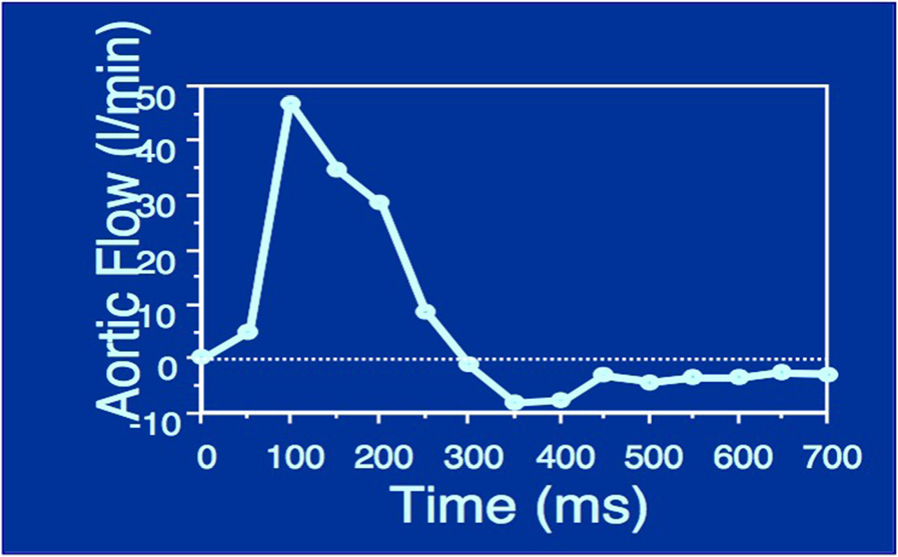
Flow versus time in the cardiac cycle for an ROI in the ascending aorta of a patient with valvular regurgitation. The negative flow in diastole is due to antegrade flow from the regurgitation. Each point represents the integrated flow over the ROI at one time point

By drawing an ROI around a vessel, basic statistics such as average velocity (Eqn. 2), peak velocity, and a velocity histogram can be determined. If ROI’s are drawn for each acquired time frame, one can determine flow as a function of time over the cardiac cycle. By plotting the flow values summed over pixels for each ROI at each time point, flow rate versus time curves can be generated (Figs. 3 and 4). The thermal noise (standard deviation) in such ROI-averaged measures can be calculated from the velocity SNR using the recently introduced approach by Hansen et al. [139]. As discussed previously, care is usually taken to trace boundaries near the edges of the vessel to eliminate contamination from other vessels and to eliminate errors in the integration due to inclusion of excess static tissue [140]. These curves are used to determine clinically relevant quantities such as aortic or pulmonary regurgitant volume, abnormal ventricular filling patterns, cardiac stroke volume (in *mL/cardiac cycle*), cardiac output (in *L/min*), and quantification of flow in left-to-right shunts [81, 141].

Time-resolved 2D PC-CMR pulse sequences with through-plane velocity encoding are typically available on all commercial MR systems. The resulting time-series of magnitude images reflect the dynamics of the underlying cardiac and/or vascular anatomy. The additionally calculated series of phase difference images represents the local velocities along the flow-encoded direction with the same spatial and temporal resolution as the anatomical data. These images are typically visualized side-by-side using gray-scale depiction of the measured blood flow velocities. Alternatively, color-overlay similar to flow visualization on Doppler echocardiography can be employed [142]. Presenting the time-series of magnitude and phase difference images in movie mode can depict the dynamics of the pulsatile flow over the cardiac cycle.

The velocity field generated by CMR can be used to determine other important physiologic parameters such as pressure gradients, vessel compliance, and wall shear stress (WSS), although these analyses are not yet available commercially to our knowledge. Pressure gradients are an indicator of the hemodynamic significance of lesions and provide unique value over anatomy and velocity alone. Pressure gradients can be determined by using the modified versions of the Navier-Stokes equations that depend on the time and spatial velocity distribution in the flow field [29, 143–145]. Note that CMR determines the spatial *gradient* of pressure, not the absolute value of pressure. The relative pressure maps generated from CMR can be visualized as color-coded static images, or time-resolved animations [145–147]. Wall shear stress, which is based on evaluating the spatial gradient of the velocity distribution at the vessel wall, has been linked to endothelial cell dysfunction and vascular remodeling [148] and the localization of atherosclerotic lesions, and can be estimated reliably from 4D flow data [149–152] given sufficient spatio-temporal resolution. Potters et al. recommend at least 8 pixels across the lumen for accurate WSS estimation, though this number may depend on the spatial interpolation (fitting) method [153].

**Fig. 5:**
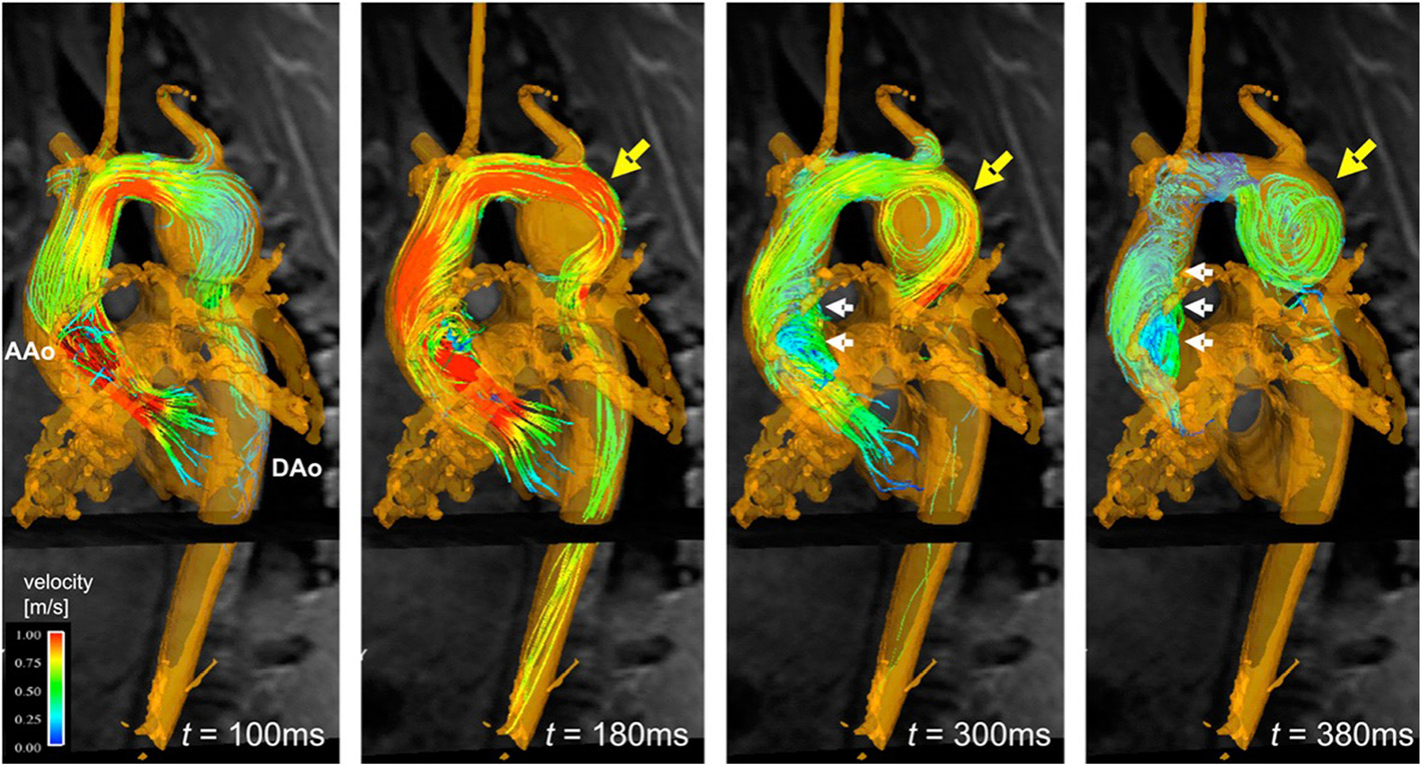
Development of vortical flow patterns in the thoracic aorta in a patient with a tubular shaped aortic arch and an aneurysm of the proximal descending aorta (yellow arrow, diameter = 4.2 cm). 3D streamlines within the 3D PC-MRA iso-surface illustrate accelerated flow along the outer aneurysm wall (t = 180 ms) and subsequent formation of a flow vortex (t = 300 ms and t = 380 ms). Note that aneurysm formation affects blood flow in the entire aorta resulting in marked helical flow in the ascending aorta (AAo, white arrows). From Ref. [107]

For the visualization of complex, three-directional blood flow within a 3D volume, various visualization tools including 2D vector-fields, 3D streamlines and time-resolved 3D particle traces have been proposed [154–156]. Since these visualization techniques have been described in other reviews (e.g., [38]), here we only briefly highlight their potential clinical importance. Figure 5 illustrates whole-heart 3D flow visualization, and shows that for a concentric aneurysm in the proximal descending aorta, a relative flow acceleration in the aortic arch developed into a flow pattern adapted to the shape of the aneurysm, i.e., highly circulating flow with a vortex core near the lateral wall [114]. Such altered flow patterns may reveal impaired flow efficiency or changes in hemodynamic parameters such as wall shear stress.

## II. Clinical applications

### Valvular heart disease

Flow assessment in the patient with valvular heart disease (VHD) seeks to answer the following questions: what is the severity of stenosis and/or regurgitation, and what are the associated anatomic abnormalities? PC-CMR may be called upon to answer these questions. While transthoracic echocardiography is invariably the first test chosen to assess flow in VHD, poor acoustic window (even with contrast administration) and limitations in acquisition plane may necessitate further testing. The incremental utility of CMR for VHD must be demonstrated in the context of other techniques such as transesophageal echocardiography (TEE), a well-established technique relied upon by surgeons to plan procedures such as mitral valve repair. TEE, which may be done intraoperative as well, readily demonstrates morphology and function of the mitral valve apparatus and other valve structures and function, albeit with risks associated with sedation and esophageal intubation. CMR may be preferable when there are contraindications to TEE, when other aspects of cardiac structure and function uniquely assessed by CMR are needed (e.g. viability), or when results from other more commonly used modalities have yielded discrepant or inconclusive results.

#### Presence and severity of stenosis

**Fig. 6:**
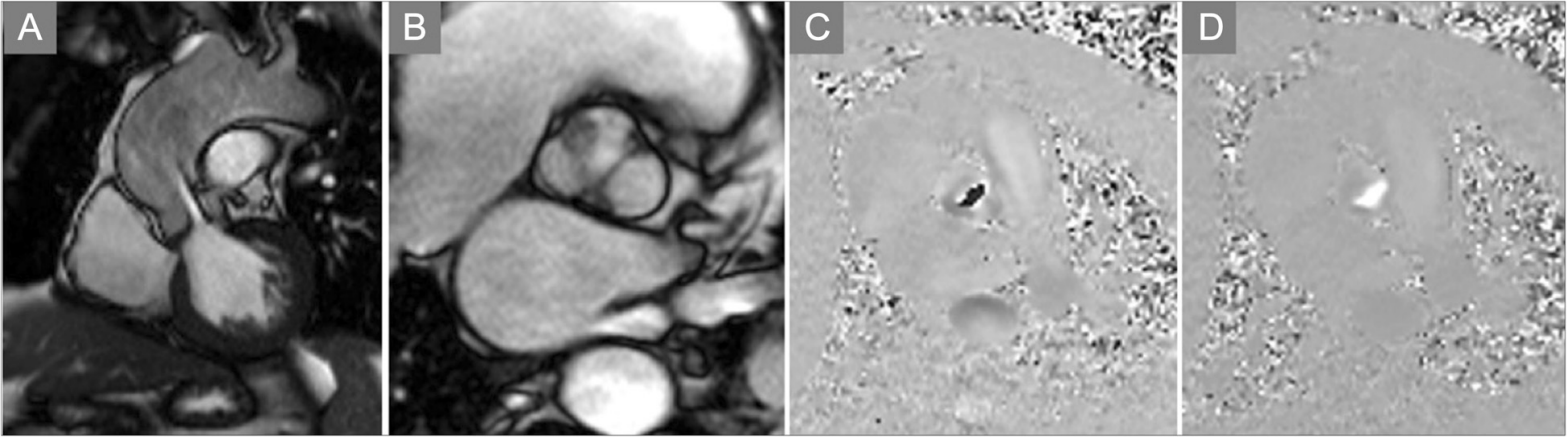
Aortic Stenosis. **a** Systolic frame from a balanced SSFP cine CMR acquisition in the left ventricular outflow tract plane shows a turbulent jet emanating from a thickened aortic valve suggesting significant stenosis. **b** Short axis view at the level of the aortic valve demonstrates a bicuspid valve en face with calcification of the anterior leaflet as well as at the commissural junctions. **c** Phase contrast image at mid-systole with VENC setting of 250 cm/s shows extensive aliasing, suggesting the peak velocity is considerably higher than 2.5 m/s. **d** Repeat phase contrast acquisition at the same location and point in the cardiac cycle with VENC increased to 450 cm/s eliminates aliasing, allowing for accurate quantification of peak velocity across this stenotic valve. See also Additional file 1

**Fig. 7:**
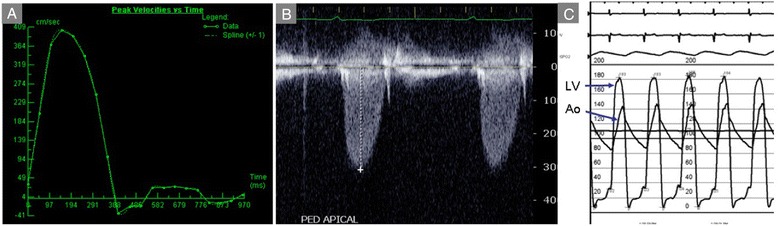
Aortic Stenosis (continued). **a** Quantification of phase-contrast data across the stenotic aortic valve shown in Fig. 6 indicates severe stenosis, with peak velocity of 4 m/s. **b** Apical continuous-wave Doppler recording in the same patient underestimates stenosis severity (3 m/s) due to misalignment relative to the direction of stenotic flow. **c** Invasive hemodynamics confirmed severe stenosis, with simultaneous aortic (Ao) and left ventricular (LV) pressure measurement translating to an aortic valve area of 0.44 cm^2^/m^2^, with <0.5 cm^2^/m^2^ considered critical AS

One of two approaches may be used to determine severity of valvular stenosis with PC-CMR. The first is to serially obtain in-plane two-dimensional velocity-encoded cine acquisitions to identify the direction of the peak velocity, and then prescribe through-plane acquisitions perpendicular to the jet direction to obtain the peak velocity. This works well when there is one predominant stenotic jet (Figs. 6 and 7, Additional file 1A-D), but fares less well in the setting of multiple jets. The other approach involves velocity encoding in multiple directions over a volume that encompasses one or more stenotic jets emanating from the valve. This may not be feasible due long acquisition times and limited availability of post-processing tools to extract the peak velocity. In practice, the former approach is most often used, recognizing that without meticulous prescriptions to identify the plane of highest velocity one may easily underestimate stenosis severity. Also, most current clinical systems use segmented techniques that yield average velocities over multiple cardiac cycles. Newer approaches to real-time velocity encoded cine imaging are preferred when available to capture beat-to-beat variation [107, 142].

Stenosis severity by PC-CMR has shown good agreement with Doppler measurements in the case of aortic stenosis (AS) [157], and smaller studies suggest agreement with catheter-derived pressure gradients [158]. Invasive pressure measurement across a severely stenotic aortic valve confers considerable mortality risk, making noninvasive estimation preferable. Further, imaging of the aorta is essential given concomitant risk of ascending aortic aneurysm that may also require repair at the time of valve surgery. CMR is thus an ideal modality for comprehensive assessment of aortic valve and associated aortopathy.

Stenosis severity by PC-CMR in other valve lesions has most commonly involved assessment of congenital pulmonic stenosis and the right ventricular outflow tract (RVOT), particularly in patients with repaired congenital heart disease where distorted RVOT geometry benefits from volumetric imaging. Mitral valve stenosis has also been measured with PC-CMR, with both peak E velocity and pressure half-time agreeing well with Doppler-echo [159].

#### Presence and severity of regurgitation

Risk of sudden death and heart failure in regurgitant valve lesions such as aortic valve insufficiency and mitral regurgitation should prompt replacement or repair of the valve when cardiac enlargement or dysfunction set in – even in the absence of symptoms [160]. The appeal of PC-CMR for measuring regurgitation severity lies in the ability to directly measure flow across a valve, e.g., regurgitant volume, instead of secondary estimates of severity such as vena contracta or proximal isovelocity surface area that are used in echocardiography. As such, PC-CMR holds particular utility when precise, serial assessment of regurgitant volume as well as ventricular response to volume overload is needed to guide timing of intervention. Regurgitation across semilunar valves is relatively straightforward in acquisition, with care taken to avoid errors due to through-plane motion of the valve plane and background phase shifts. Echocardiographers’ ratings of aortic regurgitation severity shows considerable overlap with CMR quantification of regurgitant fraction [161], underscoring the utility of the PC-CMR approach when distinguishing among AR grades of severity affects decision-making. Li and colleagues showed reasonable agreement between echo-Doppler and PC-CMR estimates of regurgitation severity in patients with repaired tetralogy of Fallot (TOF) [162]. As reviewed in the Congenital Heart Disease section below, PR quantification is an integral part of the assessment of complex post-TOF repair cardiovascular structure and function.

**Fig. 8:**
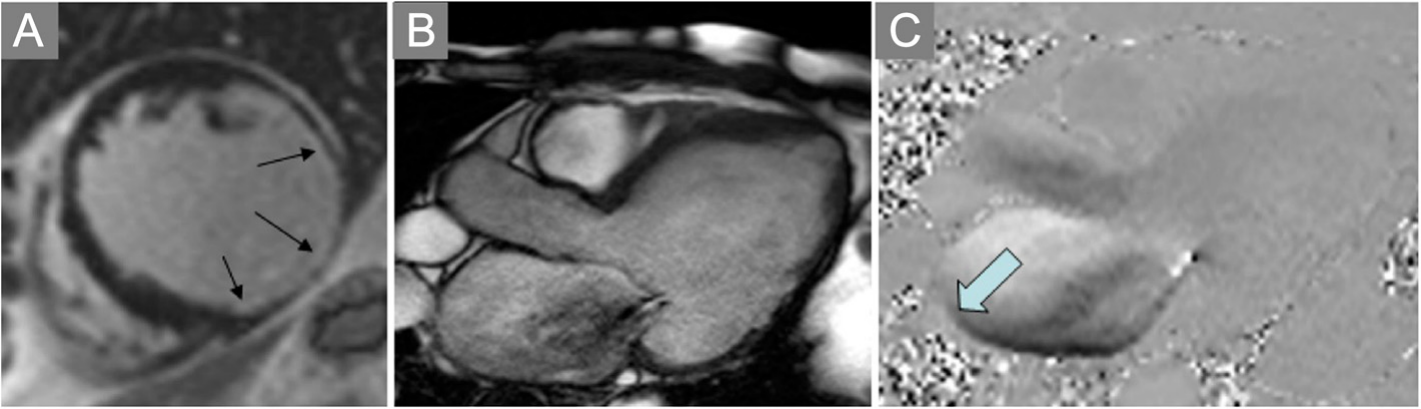
Mitral Regurgitation. **a** Late post-gadolinium enhancement imaging in a patient with dyspnea demonstrates extensive infarct scar of the inferior and lateral walls (arrows). **b** Systolic frame from a three-chamber cine SSFP acquisition shows dephasing due to turbulent mitral regurgitation flow back into the left atrium. **c** In-plane velocity-encoded cine frame in systole also demonstrates the mitral regurgitation jet (arrow), but more clearly demonstrates its eccentric direction. The jet reaches back to the pulmonary vein ostia, consistent with severe insufficiency

**Fig. 9:**
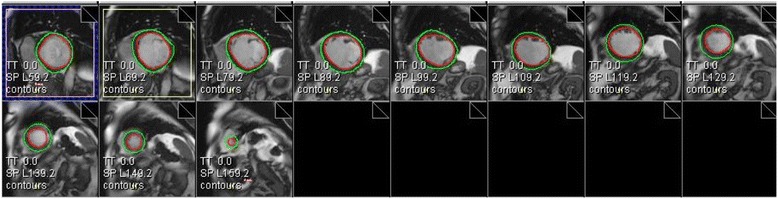
Mitral Regurgitation (continued). Quantification of mitral regurgitant volume in the case shown in Fig. 8 was done by subtracting the forward stroke volume across the aortic valve by PC-CMR from the total LV stroke volume. LV stroke volume is computed by using any of a number commercially-available software packaged (Argus, Siemens shown) to delineate endocardial borders at end-systole and end-diastole in each of the contiguous short axis cine CMR planes covering the length of the LV. The difference in volumes computed using Simpson’s rule is the LV stroke volume. In this patient, the mitral regurgitant volume was 100 (LV stroke volume by cine CMR) – 45 (aortic stroke volume by PC-CMR) = 55 mL, or a regurgitant fraction of 55 % consistent with severe mitral regurgitation

Measuring regurgitant volume across atrioventricular valves may require alternate means of estimation given the non-planar geometry of the regurgitant orifice and the often-eccentric jet directions (Figs. 8 and 9, Additional file 2). Hundley and colleagues showed that subtraction of the forward aortic stroke volume by PC-CMR from the LV stroke volume by cine CMR yielded a mitral regurgitant fraction that agreed well with angiographic estimates [163]. A recent review provided the following classification of mitral regurgitation severity as: mild = RF ≤ 15 %, moderate = RF 16–24 %, moderate-severe = RF 25–42 %, severe = RF >42 %, but also underscored the complementary utility of visually assessing MR jets on multi-slice SSFP cine imaging [80].

### Congenital heart disease

Thorough interrogation of structure and function in congenital heart disease (CHD) with CMR invariably requires flow measurement. As with VHD, Doppler-echocardiography remains widely used to detect and measure velocities and flow in CHD. Unlike VHD, however, PC-CMR may be the sole modality used in centers where expertise in congenital CMR examination affords consistent and reliable assessment of complex cardiac and vascular anatomy and hemodynamics. As detailed below, PC-CMR is an integral part of the CMR examination across a wide range of CHD lesions from simple defects to complex cyanotic heart disease.

#### Presence and severity of shunts

A variety of congenital abnormalities may lead to inappropriate transfer of blood from one side of the circulation to the other. This shunting typically occurs from the higher pressure left-sided to the lower pressure right-sided circulation, though right-to-left or bidirectional shunting may occur. The most common form of intracardiac shunt is atrial septal defect (ASD), which may produce heart failure and pulmonary hypertension due to volume overload of the right heart, with or without concomitant anomalous pulmonary venous drainage or other anomalies. ASD evaluation requires defining the presence, location, and size of the defect and surrounding rims of tissue. ASD evaluation also requires computation of the pulmonary to systemic flow ratio (Qp:Qs), for which PC-CMR is ideally suited. This is typically done by computing ratio of through-plane flow across the main pulmonary artery to through-plane flow through the proximal aortic root. A similar acquisition is used for both measurements. The time delay between these two scans is minimized in order to avoid significant changes in cardiac output between measurements. In a group of adults with ASD and other shunts referred for invasive hemodynamic evaluation, Hundley et al. validated Qp:Qs by PC-CMR against invasive oximetry and indicator dilution, showing good agreement in shunt fraction [164]. A similar comparison was made more recently by Debl et al. [165]. Beerbaum et al*.* extended this comparison to a large group of pediatric patients, confirming accuracy of Qp:Qs in both patients with as well as those without ASD [166]. This work did not find through-plane flow quantification directly across the defect as reliable, though Thomson et al*.* were able to do this successfully but only with meticulous, sequential acquisitions to obtain the ideal plane for *en face* ASD flow measurement [167].

Ventricular septal defects (VSDs) often occur in conjunction with other congenital defects such as tetralogy of Fallot or as part of atrioventricular septal defects. When PC-CMR is called upon to evaluate a VSD, it is usually in the context of complex anatomy beyond the simple muscular, restrictive VSD that produces no hemodynamic effect. As with ASDs, Qp:Qs measurement with PC-CMR that can be compared to short axis cine-derived left and right ventricular stroke volumes [168] provides a useful parameter for clinical management.

**Fig. 10:**
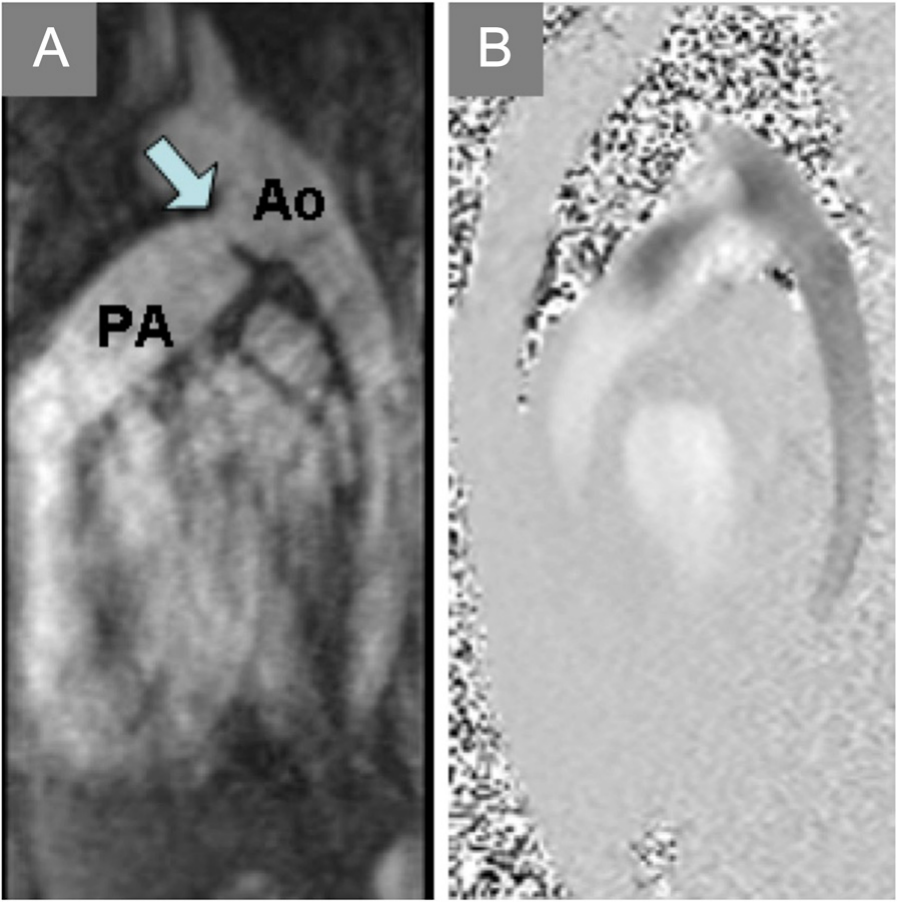
Patent Ductus Arteriosus. **a** Contrast-enhanced magnetic resonance angiogram in the sagittal plane demonstrates a patent ductus arteriosus (PDA, arrow) communicating between the proximal descending aorta (Ao) and pulmonary artery (PA). **b** In-plane PC-CMR shows flow from the aorta into the PA via the PDA

**Fig. 11:**
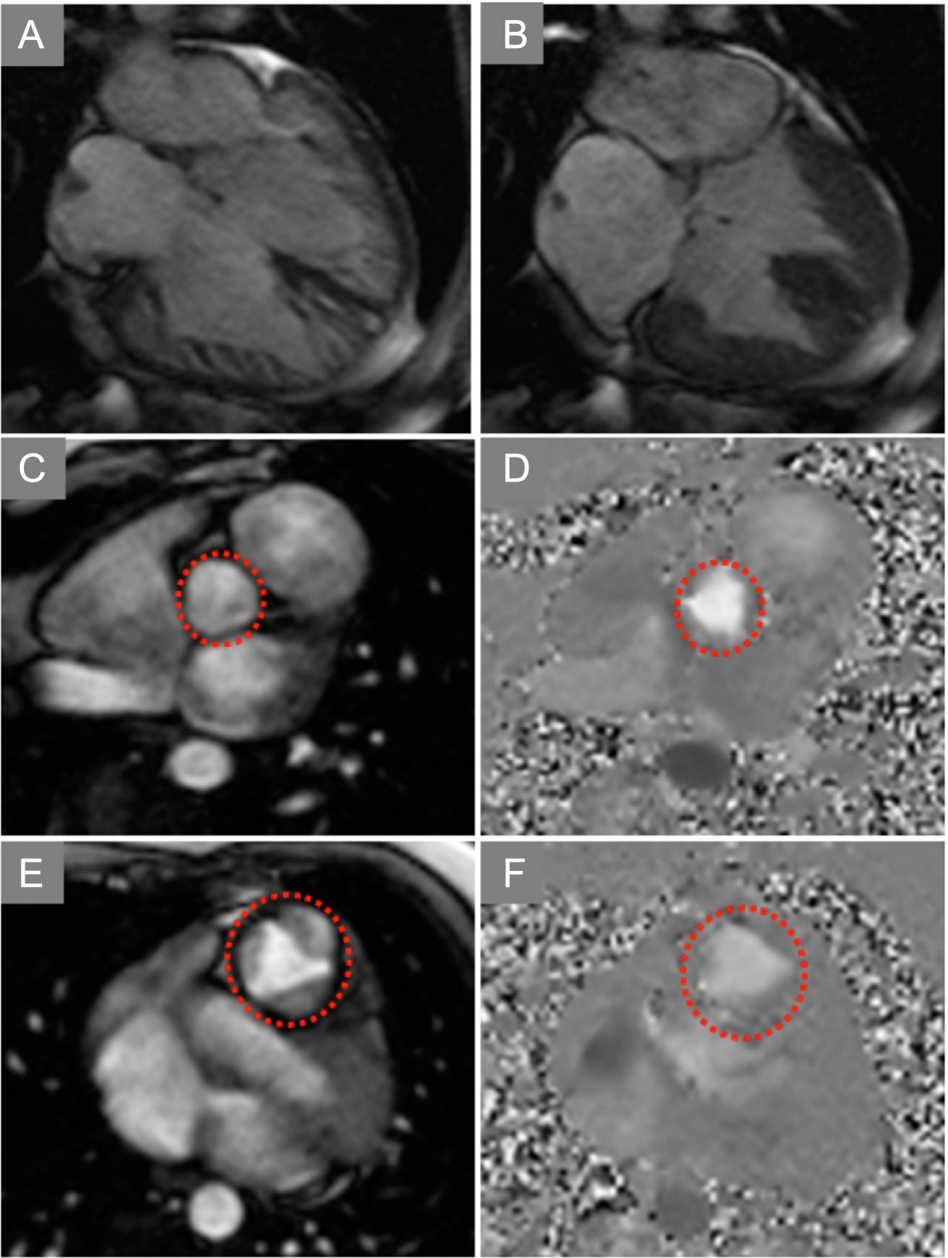
Ventricular Septal Defect. End-diastolic **(a)** and end-systolic **(b)** frames of a horizontal long-axis cine CMR acquisition demonstrate a large ventricular septal defect (VSD) of the basal half of the interventricular septum. In-plane PC-CMR showed no appreciable flow across this long-standing, restrictive VSD (Additional file 4). Through-plane PC-CMR with regions of interest (dotted circles) encircling the aortic valve **(c,d)** and pulmonic valve **(e,f)** allowed calculation of Qp:Qs that yielded a value close to 1:1, consistent with Eisenmenger physiology or advanced pulmonary hypertension limiting flow across even a large defect

Other causes of shunt flow include anomalous pulmonary venous drainage [169], patent ductus arteriosus (Fig. 10, Additional file 3), aortopulmonary collaterals (APCs) [170] and iatrogenic shunts, such as those used to palliate cyanotic heart disease. Reliance on Qp:Qs to detect and quantify shunt flow should occur in the setting of several caveats. First, advanced pulmonary hypertension may blunt left-to-right shunting (Eisenmenger physiology), particularly in the presence of a large, long-standing intracardiac defect (Fig. 11, Additional file 4). Small shunts such as those seen with intermittent flow across a patent foramen ovale may not produce significant changes from normal in Qp:Qs, and borderline abnormal Qp:Qs results should prompt a search for other evidence before inferring presence of a shunt.

#### Other congenital heart disease applications

Interrogation of branch pulmonary arteries for stenosis is an important part of the CMR examination in patients with repaired TOF. In-plane PC-CMR prescribed along the right and left pulmonary arteries can demonstrate turbulent flow and jet direction, through which perpendicular through-plane PC-CMR can be used to obtain peak velocities beyond sites of vessel narrowing that may result from prior palliative surgeries. Similarly, both in-plane PC-CMR in an RVOT view followed by appropriately-prescribed through-plane PC-CMR acquisitions yield useful qualitative and quantitative information. Distinguishing valvar from subvalvar or supravalvar pulmonic stenosis benefits from this approach, dictating distinct approaches to management. Imaging of the aorta is a central application of CMR in congenital heart disease, and PC-CMR provides complementary information on location and severity of, for instance, aortic coarctation [171].

### Flow assessment in CMR examinations

While ‘routine CMR examination’ is something of a misnomer, it is worth asking when phase contrast acquisition is required if *a priori* clinical information does not indicate a specific valvular or congenital lesion requiring flow assessment. In patients referred for myocardial viability assessment, for instance, detection of presence and severity of mitral regurgitation may be helpful in surgical planning given increased postoperative mortality in patients with significant MR whose valve disease is not addressed at the time of bypass surgery [172]. Given the increased sensitivity of PC-CMR to flow disturbances compared to cine imaging, especially short TR balanced SSFP techniques, it is reasonable to include through-plane PC-CMR at the level of mitral leaflet coaptation to screen for ischemic MR in patients referred for CMR to assess viability. In patients with right heart dysfunction of unknown etiology, screening for intracardiac shunt with first-pass perfusion imaging as well as through-plane aortic and pulmonic flow quantifications allows for estimation of Qp:Qs, with the limitations cited above in shunt detection with this approach. Finally, PC-CMR can be used to assess LV diastolic function, using a combination of mitral through-plane flow quantification and low-VENC acquisition to measure tissue velocities [173]; this combination forms a useful adjunct in comprehensive CMR examination of the patient with heart failure [173, 174].

## III. Emerging applications

### Coronary artery flow imaging

Assessment of coronary artery flow is challenging due to the small size, tortuous path, and cardiac and respiratory motion of the vessels. PC-CMR coronary flow measurements are either done with breath-holding [120, 175–178] or during free breathing [176, 178–180] with the navigator echo gating technique. Breath-holding approaches have the advantage of being fast and easy to implement, but typically provide lower spatial (>1 × 1 mm) and/or temporal (~80-100 ms) resolution than navigator gated techniques. Low temporal resolution can lead to motion blur [178], which can be especially apparent in the right coronary artery (RCA) [181] due to its extensive motion in end systole and atrial contraction. In addition, breath-hold measurements suffer from reduced filling of the ventricles leading to a reduction in coronary artery flow and heart rate [67]. In contrast, free breathing approaches do not affect patients’ hemodynamics and allow for sub millimeter spatial resolution and increased temporal resolution (~20-40 ms) [176, 178, 179] resulting in improved accuracy of flow quantification [176]. Irregular breathing patterns and diaphragmatic drift can prolong scan time and may lead to motion artifacts.

Various acquisition techniques have been employed for phase contrast coronary flow imaging including segmented fast gradient echo (2DFT) [47, 175–179], echo planar (EPI) [182], and spiral [120, 179, 183, 184]. Both EPI and spiral acquisitions allow for increased temporal resolution (~20-25 ms) while spiral data sampling also provides increased SNR, which can be traded for increased spatial resolution (0.8 × 0.8 mm) [184]. EPI only provides moderate spatial resolution (1.6 × 1.6 mm) [182].

PC-CMR coronary flow imaging has been validated against ultrasound [185, 186]^,^ Doppler flow wire [176, 180, 187] and positron emission tomography (PET) [188] measurements in animals and humans both during rest and during hyperemic stress demonstrating reasonably good correlation (r = 0.7 – 0.9) between those techniques. Coronary flow reserve measurements during hyperemic stress have shown useful for the detection of the functional significance of coronary artery stenoses and demonstrated good sensitivity and specificity for the differentiation between normal coronary segments, and segments with <75 % and >75 % luminal stenoses [180, 189]^.^ The combined measurement of coronary sinus flow and left ventricular (LV) mass allows assessment of total myocardial flow in mL/g/min as well as average coronary blood flow [190]. Validation studies with an ultrasonic volumetric flow meter in dogs showed good correlation between coronary sinus blood flow by PC-CMR and total coronary blood flow by flow meter (r = 0.98, p < 0.001) [191]. Schwitter et al*.* demonstrated good agreement between total myocardial blood flow measured by PC-CMR coronary sinus flow (divided by LV mass) and [185] N ammonia PET in healthy subjects (0.73+/-0.15 mL/g/min vs. 0.77+/-0.19 mL/g/min, r = 0.95) [188]. The clinical usefulness of this technique has been demonstrated in patients with diffuse myocardial disease such as hypertrophic cardiomyopathy [192] and in cardiac transplantation [188]. Kawada et al*.* investigated 29 patients with hypertrophic cardiomyopathy during rest and hyperemic stress [192]. Patients with hypertrophic cardiomyopathy had significantly lower myocardial blood flow during dipyridamole stress compared to healthy subjects (1.03+/-0.40 mL/g/min vs. 2.14+/-0.51 mL/g/min, p < 0.01). Schwitter et al*.* found similar results in transplant patients [188]. Coronary flow reserve was reduced in patients with transplanted hearts compared to healthy subjects (2.0+/-0.4 vs. 3.9+/-1.4, p < 0.005).

Coronary flow measurements used in concert with coronary magnetic resonance angiography, vessel wall imaging and assessment of coronary endothelial function may allow for comprehensive non-invasive assessment of coronary artery disease.

### Pulse wave velocity and vessel compliance

By making measurements at different locations along a blood vessel, PC-CMR can be used to quantify the velocity of the pulse wave generated by the ejection of blood from the left ventricle and, consequently, blood vessel compliance. The relationship between pulse wave velocity and compliance is given by

7 $$C=\frac{1}{c^2\rho }$$

where *C* is compliance, *c* is pulse wave velocity, and *ρ* is blood mass density. With the assumption of constant blood mass density, measurement of pulse wave velocity completely determines vessel wall compliance.

Structurally, vessel compliance is determined by the tissue components that comprise the blood vessel wall, including the endothelium, elastin, and collagen, and on their various amounts and interconnections. In vascular disease the various components and interconnections undergo changes and vessel compliance decreases. Decreased compliance of the aorta in particular is associated with increased risk of the progression of cardiovascular diseases such as atherosclerosis and hypertension [193]. The measurement of aortic compliance may be important in these patients and also in patients with aortic aneurysm and dissection [194].

**Fig. 12:**
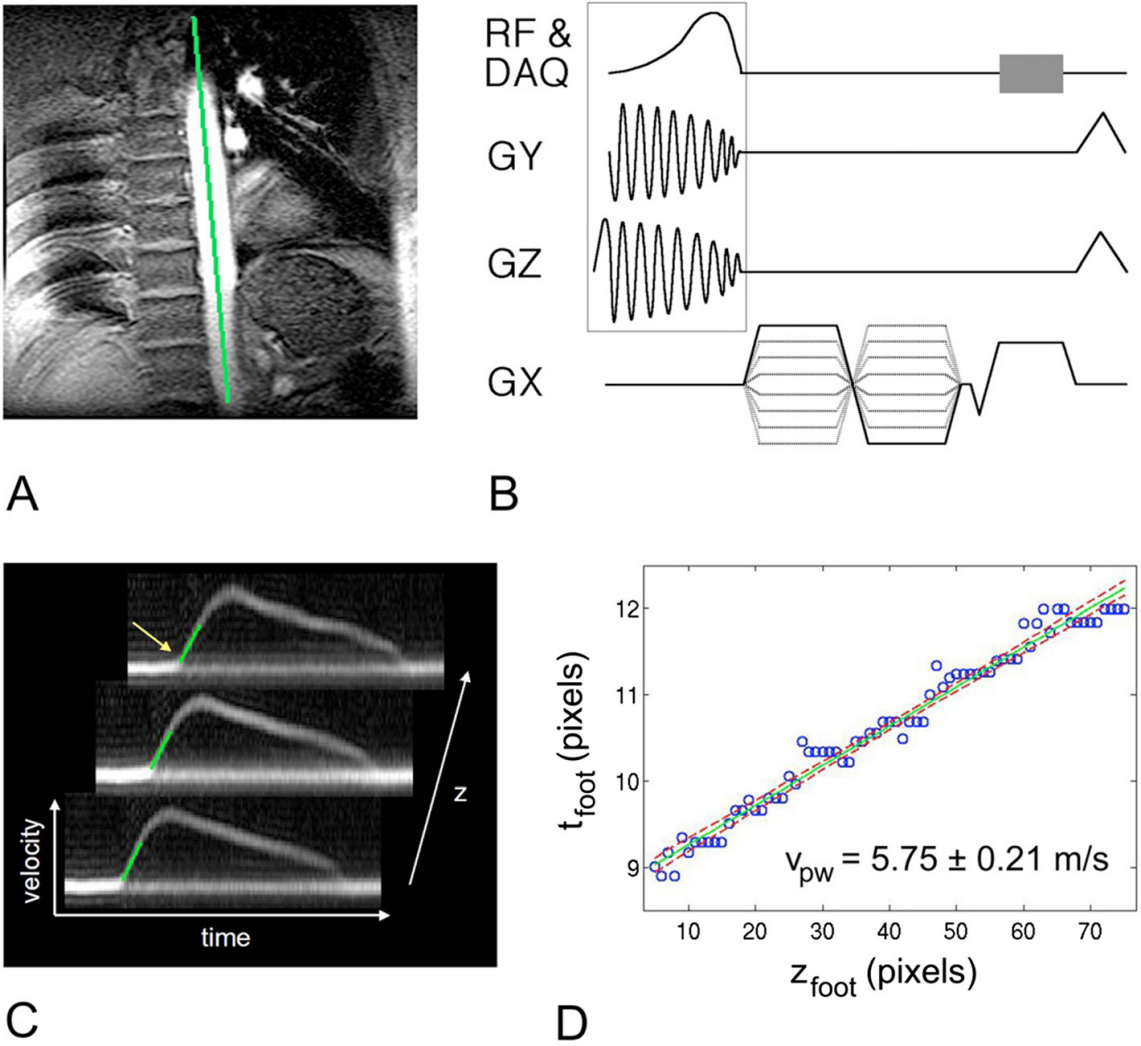
Fourier-velocity-encoded M-mode pulse sequence and pulse wave velocity data. **a** Scout image with position of M-mode pencil denoted as line. **b** ECG-gated M-mode pulse sequence, with pencil excitation (box) and Fourier velocity encoding is acquired typically over 64 heartbeats. **c** Three out of 256 velocity-vs-time waveforms generated along the length of the pencil. Green lines denote best fits to the foot of each waveform. The time of the foot (yellow arrow) is plotted against position in (d). The inverse slope of the best-fit line to the point yields the pulse wave velocity (provided by Dr. Chris Hardy, GE Global Research Center, Niskayuna, NY)

At least three variations on velocity-encoded CMR have been developed for imaging pulse wave velocity. For the first method, imaging is performed in a plane that is perpendicular to the blood vessel, images are acquired at (at least) two different spatial locations, and velocity encoding is applied in the through-plane direction [195]. If the vessel being assessed is the aorta, then a single slice positioned superior to the aortic valve can simultaneously intersect both the ascending and descending aorta. For each slice, blood flow is plotted versus time and the time corresponding to the end of the foot of the flow-time curve is identified (such as for three locations as shown in Fig. 12c). If Δ*t* is the difference in the time to the onset of flow for two slices and *D* is the distance along the centerline of the vessel between the two slices, then the pulse wave velocity (*PWV*) is

8 $$PWV=\frac{D}{\Delta t}$$

Since *PWV* is typically 4 – 10 m/sec in the aorta, phase contrast images must be acquired with a temporal resolution of around 10 ms to resolve differences in the time to the onset of flow at different slice locations. This approach has been used in a number of clinical research studies to investigate aortic compliance in aging [195], type 2 diabetes [196], cancer survivors exposed to anthracycline treatment [197], and in juvenile idiopathic arthritis [198].

Another variation on this basic method that has been applied to the aorta is to use an oblique sagittal plane that includes the axis of the vessel [199, 200]. Using this method, velocity-encoding is applied in the two orthogonal in-plane directions, rather than in the single through-plane direction. Then, the time of the onset of axial velocity (velocity down the length of the blood vessel) can be plotted versus longitudinal position along the vessel, and the slope of this line yields the pulse wave velocity. The advantage of this technique is that more points are used to estimate the slope, perhaps leading to better accuracy. The disadvantage of this method is that it only applies to vessels where a single plane can intersect the vessel over a fairly long distance.

A third method for imaging pulse wave velocity in the aorta is to use a cylindrical or “pencil-beam” radiofrequency excitation with Fourier velocity encoding [33]. This method, which is similar to M-mode echocardiography, is illustrated in Fig. 12. This technique employs a gradient for Fourier-spatial encoding of the signal along one spatial direction (the length of the aorta), and a range of velocity-encoding bipolar gradients for Fourier velocity-encoding of the signal. Two-dimensional Fourier transformation then provides velocity profiles as a function of position along the blood vessel. Because the data are not phase encoded, data acquisition is rapid compared to 2D imaging, and high temporal resolution is feasible. Once velocity profiles are measured as a function of position, pulse wave velocity is computed in a manner similar to the other techniques.

### Flow imaging to determine boundary conditions for CFD simulation

Although PC-CMR can provide reasonable and satisfactory representations of the velocity field in regions with relatively slow variations of the spatio-temporal distribution of velocities, conventional MR velocimetry is limited in regions where there are pronounced changes in velocity within a voxel. This is generally the case close to the vessel wall where there is a steep, and unknown, gradient of velocities – both in time and space [201]. It is precisely this gradient of velocities that determines the wall shear stress, the force that an individual endothelial cell experiences, and that modulates the response of the vessel wall to hemodynamic forces.

Computational fluid dynamics (CFD) has emerged as an important tool for estimation of hemodynamic descriptors that could be key indicators of the evolution of vascular disease beyond what is currently available with any non-invasive imaging method [148, 202–204]. The methodology for describing physiologically realistic intravascular flow has made rapid advances, enabled by the available computational platforms and dedicated software packages. CFD can estimate velocity fields in tortuous vessels carrying pulsatile flow with spatial and temporal resolution that is far beyond what is possible with current MR imaging approaches [205].

The governing equations that describe the flow of fluid through a prescribed geometry are the Navier-Stokes equations. Numerically solving these equations using CFD models yields the velocity field. In general, although more general approaches have been pursued, analysis of flow in vascular structures is performed with two simplifying assumptions, namely that blood can be considered to be Newtonian and that the vessel walls are rigid. Although CFD methods have been used in application to idealized representations of vascular geometry, their greatest value comes in the calculation of velocity fields on a patient-specific basis [203, 206, 207]. In order to achieve this goal, the CFD model requires accurate boundary conditions [208]. For a vascular segment of interest, the required boundary conditions are both geometric, namely a full description of the luminal surface over that segment, and physiologic, describing all time-varying flow contributions into and out of the segment of interest. CMR is the radiologic imaging modality that is best suited for defining the boundary conditions. There are a variety of MRA methods for delineating the vascular lumen, including time-of-flight MRA, contrast-enhanced MRA, and phase contrast MRA [209]. Although other modalities such as Rotational Catheter Angiography, or Multi-Detector CTA have better spatial resolution than these MR angiographic methods [210], MR is the only modality that can, in addition, provide the profile of flow velocities across the flow lumen through the cardiac cycle. Although Doppler Ultrasound has unmatched spatial and temporal resolution, it is not able to simultaneously detect for example transverse velocity components at the same time as axial velocity components, which limits its ability to estimate the velocity profile across the vessel lumen [211].

**Fig. 13:**
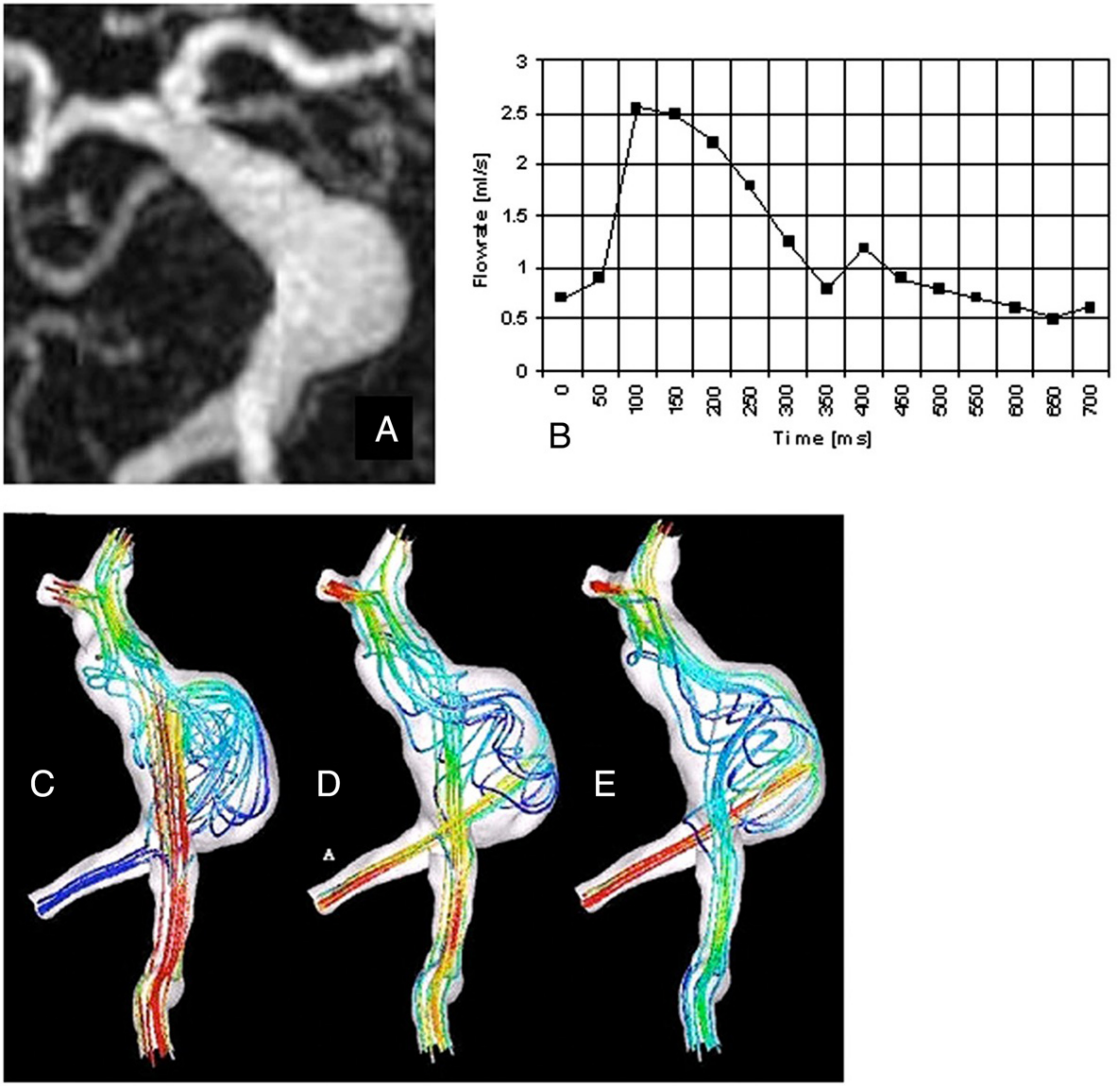
Giant fusiform basilar aneurysm: geometric and flow boundary conditions, and CFD predicted velocity fields for three different flow conditions. **a** Geometric boundaries as defined by Contrast-Enhanced MRA, **b** Flow boundary conditions from a slice transverse to a vertebral artery. **c** Velocity field with a high ratio of flow in the right vertebral artery relative to that in the left; **d** Velocity field with equal flow in each vertebral artery; and **e** Velocity field with a low ratio of flow in the right vertebral artery relative to that in the left

The importance of having an accurate estimate of the inlet flow conditions is illustrated in Fig. 13. This figure depicts the velocity fields calculated for a patient-specific geometry of an individual with a giant fusiform basilar artery aneurysm that receives flow from the two proximal vertebral arteries. The velocity field calculated for this specific geometry is shown for different assumptions on the relative flow contributions from each vertebral artery with the center image depicting equal inlet flow. It is clear that the calculated velocity fields and any derived quantity, such as WSS, vary substantially depending on the relative flow contributions from each inlet vessel.

The MR velocimetry method most often used for measuring the inlet flow velocities is two-dimensional PC-CMR applied to a slice transverse to the inlet vessel of interest [212]. If there are multiple inlet vessels, as is the case for evaluation of the velocity field in the basilar artery where both vertebral arteries serve as inlet vessels, it is then necessary to measure the flow in each vessel. Alternatively, 4D flow CMR provides a powerful approach to defining the velocities in all relevant inlet vessels that does not require the separate acquisition of velocity waveforms in each of the inlet vessels [213]. The 4D velocity data can be post-processed to determine the velocity distributions across cross-sections transverse to each of the inlet vessels and those values will then serve as boundary conditions to the CFD calculation.

In CFD, prescribing the velocity profiles across both the inlet and the outlet vessels may over-determine the system and, to the extent that the flow waveforms might be different, would present a problem that is insoluble due to violation of continuity in the domain. Rather than prescribing velocity values at the outlets, pressure values in each outlet can be set. The pressure values prescribed at the outlets define how the flow will split among the distal vessels. Another option is to specify zero streamwise velocity gradients at the outlets. In some recent studies, outlet boundary conditions were obtained by linking a relatively simple, general model of the downstream circulation to a three-dimensional, patient-specific model [214].

In principle, the complete prescription of the inlet velocity boundary conditions needed for CFD requires a detailed determination of all three components of the velocity vector in each pixel across the vascular lumen. Although this is possible, that determination comes at the cost of temporal resolution, as echoes that would otherwise provide unique points through the cardiac cycle are used to determine each additional velocity vector component. Another approach uses PC-CMR to determine the total flow through the vessel whereby only the component of the velocity vector that is perpendicular to the imaging slice is encoded. A CFD model is then constructed of the vascular geometry including inlet vessel segments proximal to the slice where the velocity was measured. The measured flow waveform is used as the inlet value at this more proximal location and a parabolic profile of velocities is assumed across the lumen. With this approach, the correct volume flow waveform is retained and the distribution of velocity vectors across the flow lumen reflects the simulated impact of the tortuosity of the proximal vessel segment.

Although CFD methods have the advantages that have been described above, namely that they are able to provide data sets with very fine resolution, important refinements are needed to correctly describe more advanced situations. These include: intermittent or turbulent flow which can occur in cases such as atherosclerotic stenoses or valvular dysfunction [215]; compliant vessel walls which pulsate through the cardiac cycle [216]; and inclusion of non-Newtonian viscosity descriptions for cases of slow recirculating flow [217]. Modifications of the standard CFD approach have been proposed for each of these cases: direct numerical simulations (DNS) requiring numerically intensive computations can account for turbulence [218]; fluid-structure interaction (FSI) approaches are being increasingly used to describe compliant vessels [219]; and there are a variety of non-Newtonian viscosity models that have been proposed [220]. These situations provide an intriguing opportunity for high resolution, multi-dimensional PC-CMR methods, offering the potential that these *in vivo* measurements could serve as a reference standard for validation of CFD approaches. The interplay of direct PC-CMR methods and numerical CFD approaches promises to be an interesting area of continued investigation.

### Tissue velocity mapping

Assessment of myocardial motion is central to the clinical evaluation of ischemia and myocardial viability. Tissue Doppler echocardiography has became an important modality for disorders involving cardiac wall motion [221], but is limited in its ability to provide reproducible and complete 3D motion estimates. CMR-based tissue tagging [222, 223] allows direct visualization of wall motion but has limited spatial resolution and requires relatively complex post-processing. PC-CMR is a potential alternative to these techniques, and offers 3D motion imaging with high spatial resolution and high reproducibility.

The application of PC-CMR to tissue velocity mapping (TVM) is in principle straightforward, however a relatively low VENC (< 5 cm/sec) is required for adequate velocity SNR. Large bipolar gradients are therefore required, which may make the need for accurate velocity offset correction particularly important in PC-based wall motion imaging. Another complication is image artifacts from flowing blood, which are also exacerbated by the low VENC. These artifacts can be suppressed using spatial presaturation (black-blood) techniques [224, 225].

Conventional 2D acquisitions [226] for myocardial phase contrast to represent the 3D structure of the heart suffer from variable (non-isotropic) spatial resolution and potential misregistration due to different long axis and short axis acquisitions typically used to obtain a full set of data. To overcome this, 4D velocity mapping can be applied in the same manner as in conventional PC-CMR [35, 227, 228]. Alternatively, a hybrid approach that combines phase contrast imaging for through-plane motion with in-plane tagging can be used to derive 3D myocardial motion [229]. To improve temporal and/or spatial resolution, the same speed-up techniques discussed previously can in principle be applied to tissue velocity mapping, including, e.g., view-sharing [25], spatio-temporal parallel imaging [125], and rapid acquisition strategies such as spiral [230].

While the acquisition technology for phase-contrast TVM can be considered mature, at least in the sense that it tracks the development of conventional PC-CMR methods, the task of estimating regional strain and motion from PC-CMR data remains an active area of research. A variety of analysis methods have been proposed, and rather than offering specific guidelines for PC-CMR TVM analysis we only provide a brief summary of suggested approaches, and of several validation studies. Among the proposed analysis methods are motion tracking and ‘glyph’ visualization methods with reduced sensitivity to errors due to noise and other sources [231, 232], and limited temporal resolution [233]. The 2008 paper by Haraldsson followed work from the same group defining a method for calculation of a time resolved strain rate tensor [234]. The Pelc group developed a number of methods for data analysis, validation, and pulse sequence design [235–238], including introduction of a closed form integration method for calculating motion trajectories from phase contrast data [239], 3D motion tracking [240], and analysis of the effects of artifacts on myocardial velocities due to flowing blood [224]. Related methods include an iterative optimization method to compute time resolved velocity maps in the myocardium [241], and an approach for Fourier tracking on time-resolved 3D phase contrast data to track ‘virtual’ markers in the myocardium [242]. The task of estimating regional strain and motion from PC-CMR data remains an active area of research.

**Fig. 14:**
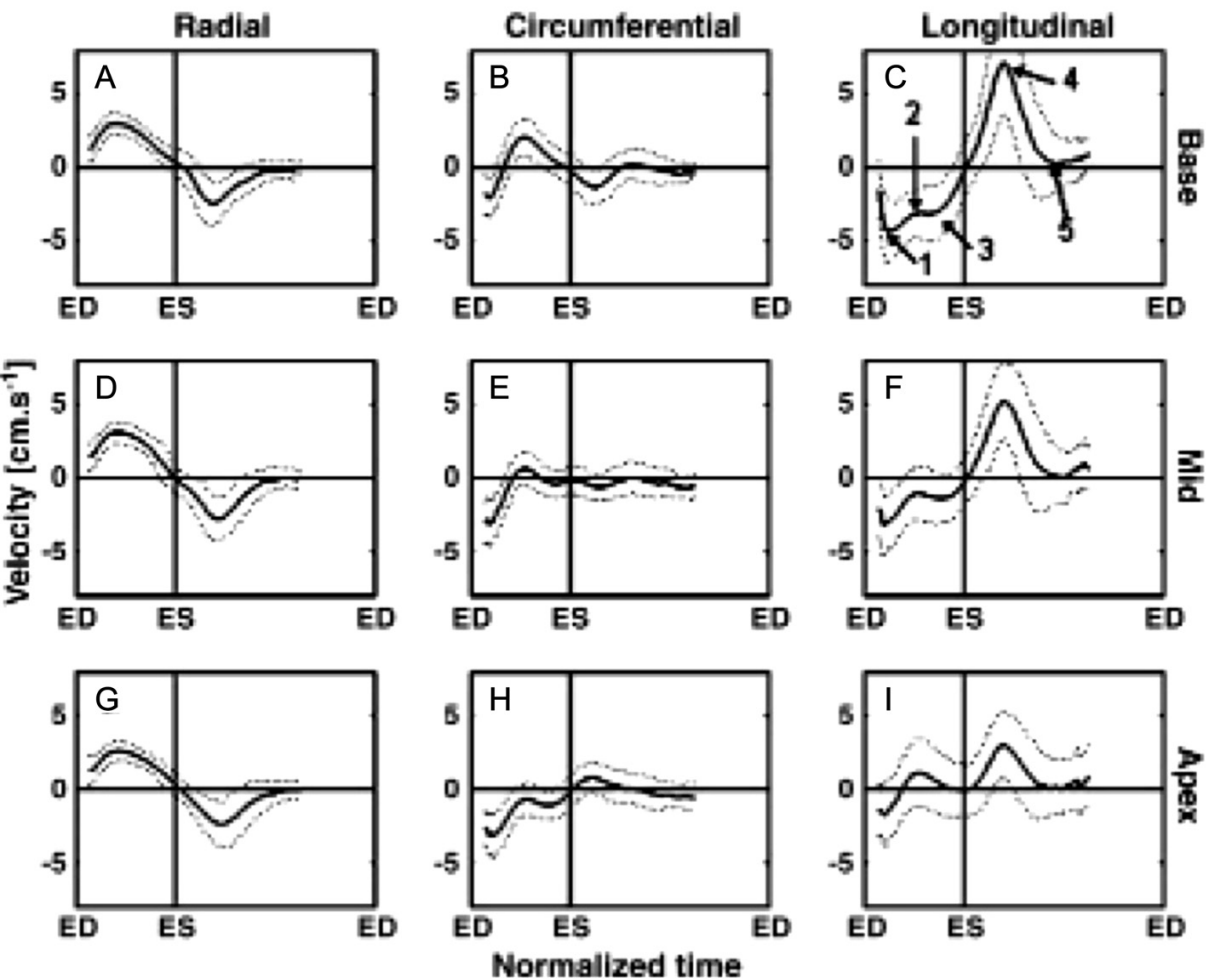
Myocardial velocity from 96 volunteers in radial, circumferential, and longitudinal directions at 3 short axis locations in the heart, normalized to the cardiac cycle length. (Reprinted with permission from Ref. [228])

A number of studies have been performed in healthy volunteers comparing strain measurements derived from PC-CMR to other methods previously published [173, 243] [244, 245], and to determine normative data from which to compare patients with cardiac abnormalities [246, 247, 248]. PC-based measures have been validated against tissue-Doppler ultrasound [173, 243] and invasive measurements [173], showing good agreement. Motion derived by phase contrast CMR has also been validated against visual inspection of signal voids caused by implanted markers [244]. Normal values of myocardial velocity were obtained by Petersen et al*.* in 96 healthy volunteers [246], using the black-blood approach of Hennig et al. Figure 14 shows typical myocardial velocity values. Age differences have also been observed. Foell et al*.* [247, 248] studied high temporal resolution myocardial velocities in different age groups in healthy volunteers and found differences in peak velocity and also in the temporal evolution of velocities across age groups.

The potential clinical usefulness of PC-CMR tissue velocity mapping has been demonstrated in several animal and human studies. Comparisons between healthy volunteers and patients include a study of long axis motion of the heart showing reduced velocities and delayed relaxation in patients [226, 249], and the work by Markl et al. [250] showing localized wall motion deficits in patients with myocardial infarction. Ischemia has been shown to cause alterations in velocity gradients in dogs [251]. Nahrendorf applied PC-CMR in addition to perfusion imaging in knockout mice, demonstrating that abnormalities in creatine kinase-deficient mice can be detected [252]. They also demonstrated in a separate study [253] that 3D myocardial motion could be obtained using phase contrast imaging at a field strength of 17.6 T in mice, following up earlier work published in 2003 [254]. Dicks et al. [255] showed the effect of coronary microembolism in a pig model on myocardial strain and compared to delayed enhancement imaging. The strain in the area at risk as determined by perfusion imaging did not differ from strain in the remote myocardium. Strain in the area of patchy microinfarction declined over a week post-infarction, providing a method of assessing longitudinal changes in myocardial function.

There have been a number of other applications of phase contrast imaging of the myocardium as well. Phase contrast sequences have been used as input to biomechanical models of the heart as shown for example by Liu [256]. Lee et al. also used phase contrast myocardial data to validate myocardial contractility modeling [257]. There have also been a number of studies using myocardial velocity as an aid to segmentation of the left ventricle [258].

Displacement ENcoding with Stimulated Echoes (DENSE) CMR is another technique to quantify myocardial motion, where phase contrast images are reconstructed and in which the signal phase is encoded for tissue displacement, as opposed to tissue velocity [259]. This is accomplished with the use of stimulated echoes, in which a component of the transverse magnetization is stored as longitudinal magnetization during an adjustable “mixing” period [260], and then refocused with a gradient lobe. While spins in both stationary and displaced tissue are fully refocused to form a stimulated echo, the *phase* of the echo (i.e., the acquired signal) will be different for stationary and displaced tissue. Therefore, the phase contrast between images acquired before and after the mixing period is directly proportional to the net tissue displacement that occurred during the mixing period. Although the use of stimulated echoes increases scan time compared to a velocity-encoded acquisition, DENSE greatly simplifies the strain calculation task. In addition, background velocity offsets are generally reduced in DENSE since identical displacement-encoding gradients are used for both images (for a given displacement-encoding direction). Compared to CMR tagging, DENSE offers higher sensitivity and spatial resolution and is becoming a popular technique for myocardial motion imaging.

### Summary

In summary, PC-CMR techniques for the measurement of velocity continue to improve through advances in equipment, pulse sequences, post-processing techniques, and visualization software. There are a number of clinical applications that use PC-CMR, most notably the evaluation of valvular diseases and flow in patients with congenital heart defects. Newer applications such as coronary artery flow measurement, tissue velocity mapping, and pulse wave velocity determination, and new methodologies such as CFD and 3D visualization, will ensure that PC-CMR techniques will expand their role in clinical and research applications.

## Additional files

Additional file 1:
**Aortic Stenosis.** **A**. SSFP cine in the LVOT plane demonstrates the turbulent jet of aortic stenosis. **B**. Small field of view cine shows a bicuspid aortic valve en face with calcification. **C**. PC-CMR acquisition at VENC setting of 250 cm/s shows aliasing that is eliminated by increasing the VENC setting to 450 cm/s, **D**. (ZIP 1337 kb) Additional file 2:
**Mitral Regurgitation.** In-plane PC-CMR acquisition in a three-chamber plane shows severe, eccentric mitral regurgitation. (ZIP 828 kb) Additional file 3:
**Patent Ductus Arteriosus.** In-plane PC-CMR acquisition in an oblique sagittal plane shows continuous flow from the aorta to the pulmonary artery via a persistent PDA. (ZIP 721 kb) Additional file 4:
**Ventricular Septal Defect.** In-plane PC-CMR acquisition in a horizontal long-axis plan with velocity encoding in the anterior-posterior direction indicates little flow across the large anatomic defect shown in Fig. 6, consistent with Eisenmenger physiology. (ZIP 82 kb)

## Abbreviations

2D: 2-Dimensional
3D: 3-Dimensional
4D: 4-Dimensional
APC: Aortopulmonary collaterals
AS: Aortic Stenosis
ASD: Atrial Septal Defect
CFD: Computational Fluid Dynamics
CFR: Coronary Flow Reserve
CHD: Congenital Heart Disease
CMR: Cardiovascular Magnetic Resonance
CTA: Computed Tomography Angiography
DENSE: Displacement Encoding with Stimulated Echoes
DNS: Direct Numerical Simulation
ECG: Electrocardiogram
EPI: Echo Planar Imaging
FLASH: Fast Low Angle Shot
FFE: Fast Field Echo
FSI: Fluid Structure Interaction
FVE: Fourier Velocity Encoding
GRAPPA: Generalized Autocalibrating Partially Parallel Acquisition
LV: Left Ventricle
MR: Mitral Regurgitation
MRA: Magnetic Resonance Angiography
MRI: Magnetic Resonance Imaging
NMR: Nuclear Magnetic Resonance
PC: Phase Contrast
PC-CMR: Phase Contrast Cardiovascular Magnetic Resonance
PET: Positron Emission Tomography
PWV: Pulse Wave Velocity
RCA: Right Coronary Artery
ROI: Region of Interest
RVOT: Right Ventricular Outflow Tract
SENSE: Sensitivity Encoding
SPGR: Spoiled Gradient Echo
SNR: Signal to Noise Ratio
SSFP: Steady State Free Precession
TE: Echo Time
TEE: Trans-esophageal Echocardiography
TR: Repetition Time
VENC: Velocity Encoding
VHD: Valvular Heart Disease
VSD: Ventricular Septal Defect
WSS: Wall Shear Stress
